# Supplementary material for: Spatial inhomogeneity and temporal dynamics of a 2D electron gas in interaction with a 2D adatom gas
Source: Sci Rep. 2017 Sep 6;7:10642. doi: 10.1038/s41598-017-10300-6 (PMC5587567; doi:10.1038/s41598-017-10300-6)
Supplement: Supplementary file 1 — supplementary information [file 41598_2017_10300_MOESM1_ESM.pdf]

Spatial inhomogeneity and temporal dynamics of a 2D electron gas in interaction with a 2D adatom gas

F. Cheynis[1,*], S. Curiotto[1], F. Leroy[1,], and P. Müller[1]

[1]Aix Marseille Univ, CNRS, CINAM, Marseille, France
[*]cheynis@cinam.univ-mrs.fr

Low-energy electron microscopy (LEEM) & work function measurements

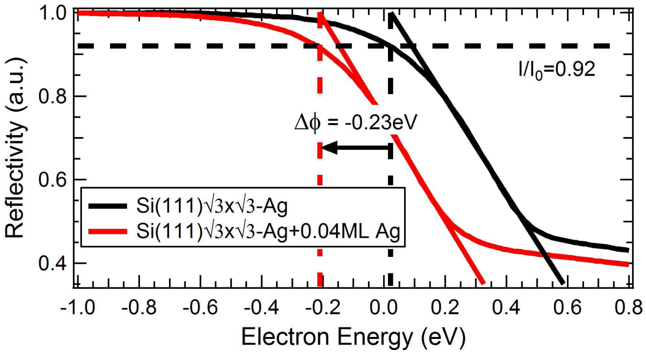

Figure S1. Intensity-Incident electron beam energy $I(\varepsilon)$-reflectivity curve of a $\sqrt{3}\times\sqrt{3}$-Ag surface at 220 K (black curve). Upon a 0.04 ML-Ag deposition at 220 K (red curve), the $I(\varepsilon)$-curve shows a lowering of the electron injection threshold of $\Delta\phi = -0.23\,\mathrm{eV}$.

LEEM & adatom concentration monitoring

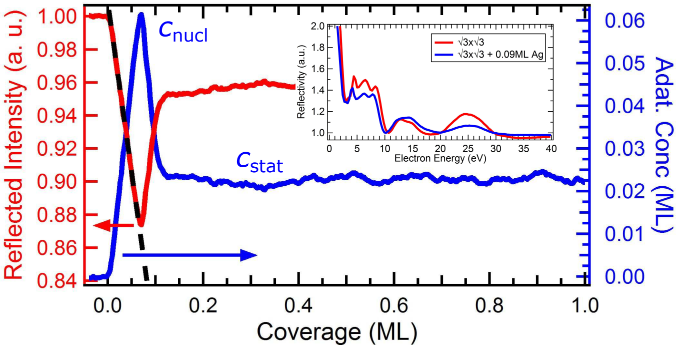

Figure S2. Surface reflectivity change induced by a Ag-2DAG deposition at RT (red curve) and the deduced Ag-2DAG concentration monitoring (blue curve, see text for details). The dashed dark line is a linear fit used to derive the value of Σ[1]. The inset shows the reflectivity curves of a $\sqrt{3}\times\sqrt{3}$-Ag surface before and after a 0.09 ML Ag-deposition at 220 K. A clear sensitivity to the deposition is observed at 24 eV.

Supplementary Video S1

Supplementary Video S1 is a low-energy electron microscopy movie (.avi) showing the Si(111)$\sqrt{3} \times \sqrt{3}$-Ag surface during a Ag-2DAG deposition at 226 K for two electron beam energies. In real time, the movie lasts 20 min. At ε=24 eV, the image shows the Ag-2DAG concentration variations. At ε=1.8 eV, where the LEEM image is more sensitive to the surface electrostatic potential, the movie illustrates qualitatively the surface work function time evolution. This evidences the spatial inhomogeneity and the temporal dynamics of the 2D electron gas obtained below room-temperature induced by the distribution variations of the Ag-2D adatom gas resulting from the growth of a Ag 3D phase and by the occurrence of an Ehrlich-Schwoebel diffusion barrier of 150$\pm$10 meV (see manuscript text for details).

References

1. Farias, D. & Rieder, K.-H. Atomic beam diffraction from solid surfaces. *Rep. Prog. Phys.* **61**, 1575 (1998). DOI 10.1088/0034-4885/61/12/001.
